# Strongly coupling Cu with MoP for high-efficiency electrochemical nitrate-to-ammonia conversion and zinc-nitrate battery applications

**DOI:** 10.3389/fchem.2025.1629904

**Published:** 2025-07-17

**Authors:** Chen Yang, Yuanyuan Chen, Zhimin He, Rong Li, Xinglong Gou

**Affiliations:** ^1^ Precise Synthesis and Function Development Key Laboratory of Sichuan Province, College of Chemistry and Chemical Engineering, China West Normal University, Nanchong, China; ^2^ Sichuan University of Arts and Science, Dazhou, China

**Keywords:** copper nanoparticles, molybdenum phosphide nanoparticles, heterostructure, nitrate reduction reaction, Zn-nitrate battery

## Abstract

The electrochemical nitrate reduction reaction (NITRR) offers a sustainable route for ammonia synthesis and environmental remediation but faces challenges such as sluggish kinetics and competing hydrogen evolution. This study aims to address these limitations by designing a Cu/molybdenum phosphide (MoP) heterostructure catalyst through one-pot calcination, which integrates Cu nanoparticles with MoP nanograins. Structural and electronic analyses confirm the formation of intimate Cu–MoP interfaces, where charge redistribution polarizes Cu to an electron-deficient state (Cu^δ+^) and enriches MoP with electrons. This configuration enhances nitrate adsorption on Cu^δ+^, while MoP efficiently supplies protons via accelerated water dissociation. The Cu/MoP catalyst achieves a record-high NH_3_ Faradaic efficiency (FE) of 98.93% and a yield rate of 30.72 mmol h^−1^ cm^−2^ at −0.5 V (vs. RHE), outperforming isolated Cu or MoP. When deployed in a Zn-nitrate battery, the composite cathode delivers a peak power density of 12.97 mW cm^−2^. This work provides a promising solution to the insufficient active hydrogen supply and poor NH_3_ conversion efficiency of Cu-based nitrate reduction catalysts.

## Graphical Abstract

**Figure:**
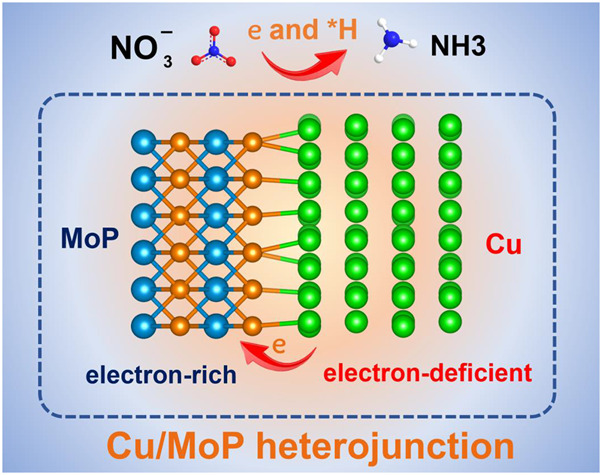
Strongly coupling Cu with MoP produces abundant Cu and MoP heterostructure interfaces and leads to electron-deficient Cu and electron-rich MoP, which synergistically promote NITRR activity and zinc-nitrate battery performance.

## 1 Introduction

The electrochemical nitrate reduction reaction (NITRR) offers a dual solution to environmental and energy challenges by converting nitrate pollutants into valuable ammonia (NH_3_), a critical feedstock for fertilizers and a carbon-free energy carrier (Zhang et al., 2022; Zhou and Sun, 2022; Fu et al., 2023). Traditional NH_3_ production via the Haber–Bosch process is energy-intensive, accounting for ∼1%–2% of global energy consumption and relying on fossil fuel-derived hydrogen (Cheng et al., 2018; Ma et al., 2020; Sun et al., 2021). In contrast, NITRR powered by renewable electricity operates under ambient conditions and utilizes nitrate—a widespread water contaminant from agricultural and industrial activities—as the nitrogen source (Atrashkevich et al., 2022; Xiong et al., 2024). However, the NITRR involves a complex eight-electron/nine-proton transfer process (NO_3_^−^ + 9H^+^ + 8e^−^→ NH_3_ + 3H_2_O), which suffers from sluggish kinetics, a competing hydrogen evolution reaction (HER), and byproduct formation (e.g., NO_2_^−^ and N_2_), leading to low NH_3_ selectivity and Faradaic efficiency (FE) (Yao et al., 2019; Fu, 2023; Yu et al., 2024). These challenges stem from the difficulty in synchronizing proton-coupled electron transfers and stabilizing multiple intermediates (Fan et al., 2022). Developing catalysts that balance nitrate adsorption, proton supply, and intermediate energetics is thus critical to advancing NITRR toward practical applications.

Copper-based catalysts are widely explored for the NITRR due to their strong affinity for nitrate adsorption and moderate activity (Ullah et al., 2023; Yin et al., 2024; Zhu et al., 2025). The *d*-orbital electrons of Cu facilitate nitrate binding, while its redox flexibility enables stepwise reduction (Xie et al., 2025). However, Cu faces the following intrinsic limitations: (1) excessive HER competition at high overpotentials due to insufficient *H coverage for NH_3_ synthesis, (2) poor selectivity toward NH_3_ as intermediates like NO_2_ desorb prematurely, and (3) rapid deactivation from surface oxidation or poisoning (Zhou et al., 2023; Liu et al., 2024; Liu et al., 2025). To address these issues, strategies such as alloying, heteroatom doping, and heterostructure engineering have been employed (Liu et al., 2022; Geng and Ji, 2024; Li Y. et al., 2025). For instance, copper–palladium alloy/CuO heterostructure (CuPd/CuO) offers a more thermodynamically favorable NHO route than the isolated CuPd alloy, benefiting from the synergetic optimization of CuPd and CuO for water dissociation and the NITRR (Li J. et al., 2025). These approaches highlight the critical role of proton supply in an efficient NITRR process, which requires not only optimized active sites for nitrate activation but also a robust proton source to sustain the multi-step hydrogenation process. Integrating Cu with proton-rich co-catalysts to decouple HER competition and enrich active sites of N-intermediate is very promising strategy to boost nitrate-to-ammonia conversion efficiency.

Molybdenum phosphide (MoP), a highly active HER catalyst, may present a unique opportunity to enhance Cu-based NITRR. MoP exhibits strong water dissociation capability, generating abundant protons while suppressing H_2_ evolution due to its near-optimal hydrogen binding energy, which holds great promise for the hydrogenation of NO_3_^−^ to NH_3_ (Gu et al., 2021; Zhang et al., 2021; Guo et al., 2024). Moreover, the semi-metallic nature of MoP is favorable for regulating the electronic structure of other materials upon the heterojunction construction. Different electronegativity values of counterparts in the MoP-based heterostructure can induce charge redistribution at the interface (Gu et al., 2021). For instance, Liu et al. (2020) revealed that coupling α-MoC_1-x_ with MoP can induce strong electronic interactions at the α-MoC_1-x_–MoP interface, leading to moderate free energy of H adsorption and improved HER activity. Thus, a Cu/MoP heterostructure could synergistically address the limitations of standalone Cu catalysts by combining efficient nitrate reduction on Cu with proton generation and electronic modulation from MoP.

In this study, a Cu/MoP composite was synthesized via one-pot calcination, forming intimate heterojunctions between Cu nanoparticles and MoP nanograins. Structural characterization confirms the successful construction of the abundant Cu and MoP interface, and the interface induces the formation of electron-deficient Cu and electron-rich MoP, which synergistically facilitate the NITRR process and active hydrogen generation. The optimized Cu/MoP composite achieves a record NH_3_ FE of 98.9% and a yield rate of 30.72 mmol h^−1^ cm^−2^ at −0.5 V vs. RHE, outperforming isolated Cu or MoP. When deployed in a Zn-nitrate battery, the Cu/MoP cathode delivers a high open-circuit voltage of 1.35 V and a peak power density of 12.97 mW cm^−2^, demonstrating its dual functionality in energy storage and environmental remediation.

## 2 Results and discussion

**FIGURE 1:**
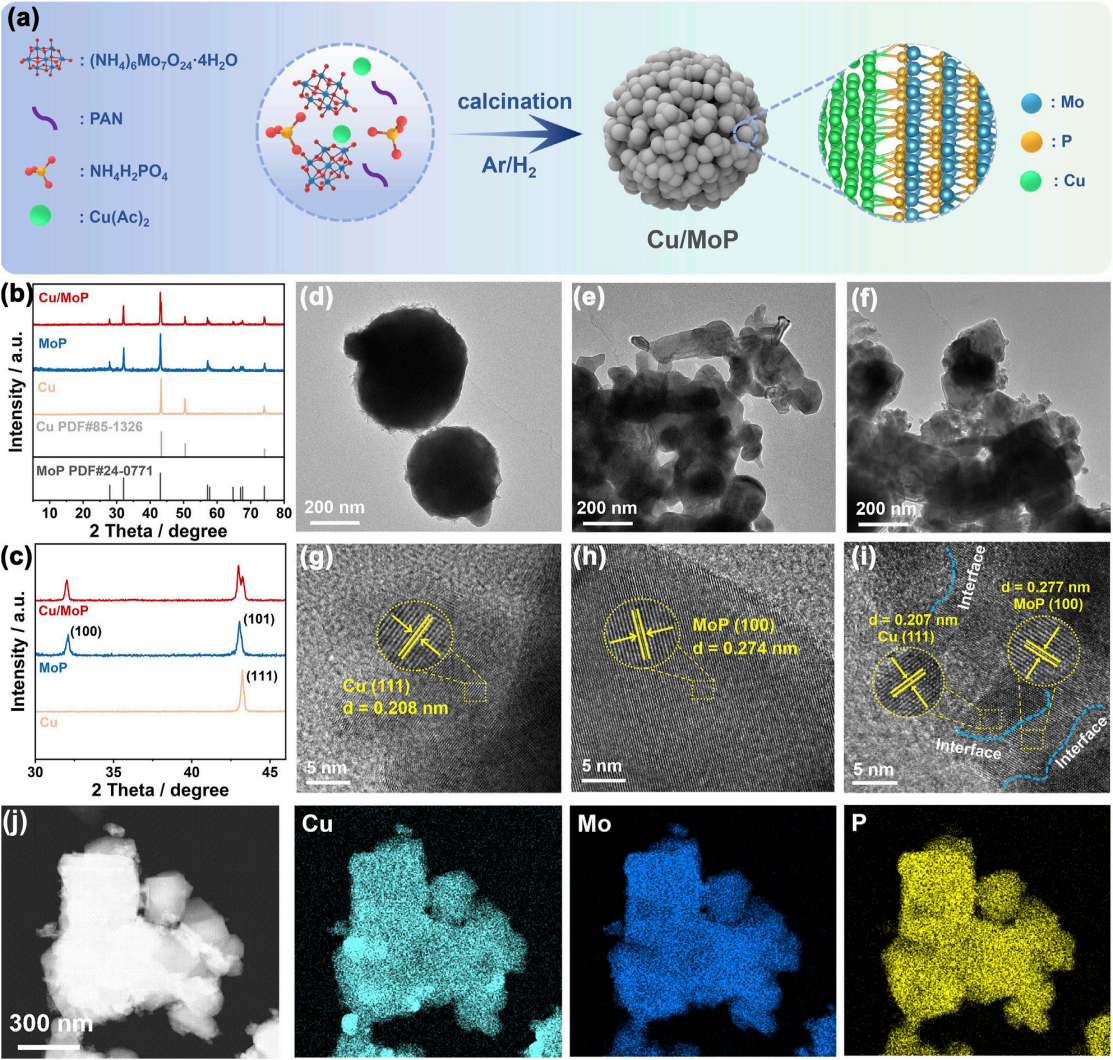
**(a)** Schematic illustration of the synthesis pathway of the Cu/MoP composite; XRD patterns **(b)** and the partially enlarged diffraction peak **(c)**; TEM **(d–f)** and HR-TEM **(g–i)** images of Cu **(d,g)**, MoP **(e,h)**, and Cu/MoP **(f,i)**; **(j)** STEM-HAADF image of Cu/MoP.

The schematic illustration of the synthesis of the Cu/MoP composite is shown in Figure 1a. (NH_4_)_6_Mo_7_O_24_⋅4H_2_O, NH_4_H_2_PO_4_, and Cu(Ac)_2_ were employed as Mo, P, and Ce sources, respectively. These precursors were thoroughly ground and then calcined at 800°C in an Ar/H_2_ atmosphere to produce the Cu/MoP composite. In addition, PAN was particularly used to avoid grain growth and promote the formation of nanoparticles The isolated Cu or MoP samples were prepared by the same method, except for the former was synthesized without (NH_4_)_6_Mo_7_O_24_⋅4H_2_O and NH_4_H_2_PO_4_ and the latter without Cu(Ac)_2_, in their respective synthesis processes.

Powder X-ray diffraction (XRD) characterization was utilized to study the phase composition and crystal structure of the as-obtained samples. As shown in Figures 1b,c, the as-prepared Cu/MoP composite has the typical crystal structure, and the corresponding diffraction peaks located at 27.86°, 32.02° and 42.98° can be, respectively, indexed to the diffraction patterns of (001), (100), and (101) planes of MoP phase (PDF#24-0771), while peaks at 43.24° and 74.30° were, respectively, assigned to the (111) and (220) planes of the Cu phase (PDF#85-1326), which suggests the presence of both Cu and MoP phases in the Cu/MoP composite (Kibsgaard and Jaramillo, 2014; Liu et al., 2023). Meanwhile, the XRD patterns proved that the isolated Cu or MoP with pure phase was successfully prepared. To investigate the effect of Cu content in the Cu/MoP composite on catalytic activity, two other samples, namely, Cu/MoP-5 and Cu/MoP-15, were also synthesized. As shown in Supplementary Figure S1, the same XRD patterns as the Cu/MoP sample were also detected in Cu/MoP-5 and Cu/MoP-15, indicating that all Cu/MoP samples contain Cu and MoP phases. Furthermore, normalized XRD patterns are shown in Supplementary Figure S1c. The content of the Cu phase increases in the following order: Cu/MoP-15 > Cu/MoP > Cu/MoP-5. Due to the different roles of Cu and MoP phases in electrocatalytic NITRR processes, increased Cu content may show significant effects on catalytic performance. Field-emission scanning electron microscopy (FE-SEM) and field-emission transmission electron microscopy (FE-TEM) were used to investigate the nanostructure and morphology of the as-prepared samples. As shown in Supplementary Figure S2; Figures 1d–f, these three samples, namely, Cu, MoP, and Cu/MoP, all exhibit aggregated nanoparticles, and the Cu/MoP composite displays a grain size similar to that of the isolated MoP sample and smaller than that of the isolated Cu sample, indicating that coupling MoP with Cu nanoparticles can effectively refine the grain size. This effect can be attributed to the different crystal structures and grain growth rates of Cu and MoP. High-resolution TEM (HR-TEM) images (Figures 1g–i) reveal that these three samples have a typical crystal structure. The observed interplanar spacing of lattice fringes is 0.274 nm for the isolated MoP and 0.208 nm for the bare Cu, which can be indexed to the (100) plane and (111) plane of MoP and Cu, respectively. For the Cu/MoP composite, both of the above-mentioned planes are observed, and abundant Cu and MoP heterojunction interfaces (indicated by blue dotted lines) are present, suggesting good coupling between Cu and MoP nanoparticles. Figure 1j shows the scanning transmission electron microscopy with high-angle annular dark-field (STEM-HAADF) images of Cu/MoP, and the corresponding elemental mapping images demonstrate the uniform distribution of Cu, Mo, and P, further confirming the uniform distribution of Cu and MoP nanograins in the Cu/MoP composite.

**FIGURE 2:**
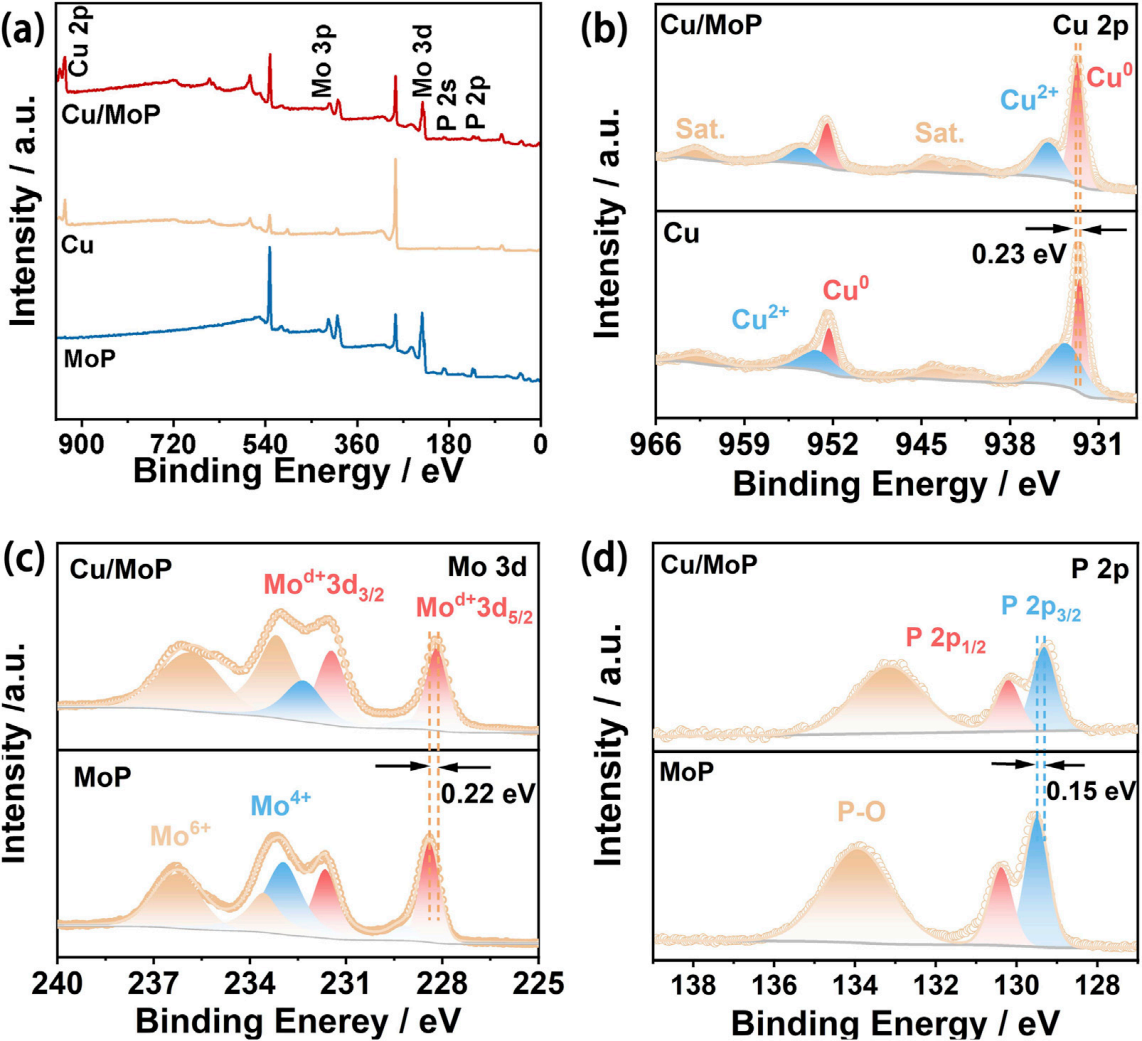
**(a)** XPS survey spectra of Cu/MoP, Cu, and MoP samples; **(b)** high-resolution Cu 2p spectra of Cu/MoP and Cu; high-resolution Mo 3d **(c)** and P 2p **(d)** spectra of Cu/MoP and MoP.

To characterize the surface electronic state and chemical composition of the as-prepared samples, X-ray photoelectron spectroscopy (XPS) was conducted. The survey spectrum of the Cu/MoP sample (Figure 2a) confirms the existence of Cu, Mo, and P in the composite, which are assigned to the Cu and MoP phases. However, the signals of Mo and P are not detected in the Cu sample, while the signal of Cu is not detected in the MoP sample, revealing the successful preparation of isolated Cu and MoP materials. High-resolution Cu 2p spectra of the isolated Cu and Cu/MoP samples are shown in Figure 2b. For the bare Cu sample, a pair of binding energies located at 932.40 and 952.31 eV are attributed to the 2p_3/2_ and 2p_1/2_ of Cu^0^, respectively, while two peaks positioned at 933.79 and 953.63 eV are assigned to Cu^2+^, respectively (Lin et al., 2024). However, the binding energies of Cu^0^ of the Cu/MoP composite are centered at 932.63 and 952.54 eV, showing a 0.23 eV positive shift compared to the isolated Cu nanoparticles. In high-resolution Mo 3d spectra of MoP nanoparticles (Figure 2c), the binding energies of Mo^δ+^ 3d_5/2_ and Mo^δ+^ 3d_3/2_ are located at 228.45 eV and 231.65 eV, which are ascribed to Mo-P species. The doublets at 233.60/236.30 eV and 229.25/232.95 eV are assigned to the Mo^6+^ (MoO_3_) and Mo^4+^ (MoO_2_) species, respectively (Wei et al., 2021). For the Cu/MoP composite, the doublet peaks of Mo^δ+^ are positioned at 228.23 eV (Mo^δ+^ 3d_5/2_) and 231.43 eV (Mo^δ+^ 3d_3/2_), manifesting a 0.22 eV negative shift of binding energy compared to the isolated MoP nanoparticles. Furthermore, the high-resolution P 2p spectra (Figure 2d) can be deconvoluted into three peaks. In MoP, the P 2p_3/2_ and P 2p_1/2_ of Mo-P species, respectively, center at 129.50 eV and 130.40 eV, while the peak assigned to P_2_O_5_ is located at 134.00 eV. However, the binding energies of P 2p_3/2_ and P 2p_1/2_ in Cu/MoP negatively shift to 129.35 and 130.25 eV, respectively. These results suggest that the Cu and MoP interface has strong interactions, and electrons localized on the Cu surface in the Cu/MoP composite are transferred to the MoP surface, which produces a charge-polarized Cu^δ+^ (0 < δ < 1) chemical environment and an electron-rich MoP surface. The electron-deficient Cu active sites may promote the production of active hydrogen species (*H) and facilitate the NITRR, and electron-rich MoP surface can strengthen H bonding, which may supply abundant *H for NITRR processes.

**FIGURE 3:**
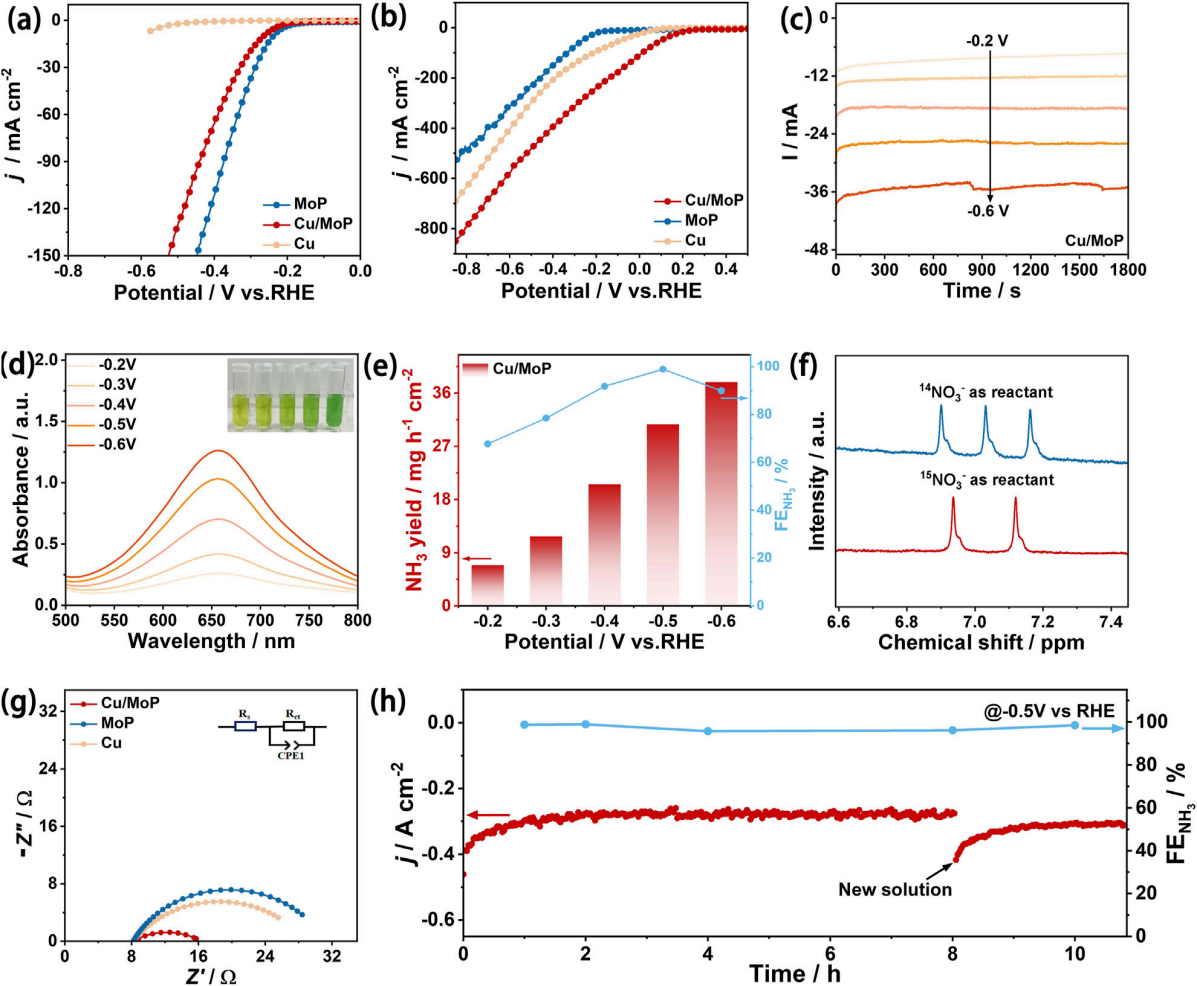
Electrochemical NITRR performance evaluation. **(a)** LSV curves of Cu, MoP, and Cu/MoP samples in 1.0 M KOH electrolyte without NaNO_3_; **(b)** LSV curves of Cu, MoP, and Cu/MoP samples in the mixed electrolyte of 1.0 M KOH and 1.0 M NaNO_3_; **(c)** chronoamperometry tests of Cu/MoP for 0.5 h at different applied potentials from −0.2 to −0.6 V; **(d)** UV-Vis absorption spectra of the product solution after chronoamperometry tests of Cu/MoP (the inset is the digital photograph of the product solution stained with the indophenol blue indicator); **(e)** NH_3_ faraday efficiency and yield rate of Cu/MoP; **(f)** ^1^H-NMR spectra of the electrolyte after NITRR electrolysis at −0.5 V using Na^15^NO_3_ or Na^14^NO_3_ as the N source. **(g)** Nyquist plots of MoP, Cu, and Cu/MoP at a applied potential of −0.4 V; **(h)** I–t test of Cu/MoP at −0.5 V and the corresponding FEs.

The electrocatalytic NITRR performance of the as-obtained catalysts was evaluated in an H-type cell using a three-electrode system, where the cathode and anode compartments were separated using a commercial Nafion 117 membrane. First, the linear sweep voltammetry (LSV) tests were conducted to assess the catalytic activity of the as-prepared samples. Figure 3a shows the LSV curves of Cu, MoP, and Cu/MoP in 1.0 M KOH electrolyte without NaNO_3_, and the corresponding current response is attributed to HER processes. These results show that MoP and Cu/MoP have certain catalytic HER activity in NaNO_3_-free electrolyte, and the overpotential of MoP is 239 mV at a current density of 10 mA cm^−2^, while that of the Cu/MoP composite is 265 mV, indicating that H_2_ release occurs more readily on MoP nanoparticles than on the Cu/MoP composite. However, the isolated Cu nanoparticles are inert to HER, suggesting insufficient *H coverage. Interestingly, in the mixed electrolyte of 1.0 M KOH and 1.0 M NaNO_3_, the current response of these three samples is significantly enhanced, which is ascribed to the NITRR processes. Under the applied voltage window from −0.1 V to −0.8 V, the polarization current density values of these three catalysts are in the following order: Cu/MoP > Cu > MoP, and the current density of Cu/MoP is up to 482 mA cm^−2^ at −0.5 V, indicating its highest NITRR catalytic activity. According to the LSV curves of HER and NITRR, although the bare MoP has good catalytic capacity for water dissociation and active hydrogen generation, excessive H_2_ evolution may lead to insufficient *H retention for the conversion of nitrate to ammonia. Although Cu is widely reported as an effective catalyst for the NITRR, the conversion efficiency suffers from insufficient active hydrogen (Ullah et al., 2023). Our results also prove this conclusion. The unsatisfied NITRR catalytic activity of isolated Cu is attributed to its poor catalytic ability for water dissociation, which is unfavorable for active hydrogen generation. Therefore, coupling Cu and MoP overcomes their respective shortcomings. At the Cu and MoP heterojunction, the Cu phase provides highly active sites for the NITRR, while the MoP phase offers abundant active hydrogens. Furthermore, strong electron transfer from Cu to MoP produces the electron-deficient state of Cu and the electron-rich state of MoP, which synergistically promote the transformation of nitrate and the generation of active hydrogen. To further evaluate the NITRR catalytic activity of the Cu/MoP composite, LSV curves at different NO_3_^−^ concentrations were also recorded (Supplementary Figure S3), which reveals that the Cu/MoP composite exhibits excellent NITRR catalytic activity even at low NO_3_^−^ concentrations in the electrolyte. In addition, to investigate the effects of different Cu contents of the Cu/MoP composite on the catalytic activity, Cu/MoP-5 and Cu/MoP-15 were also studied in NO_3_^−^ and NO_3_^−^-free electrolytes. As shown in Supplementary Figure S4a, the HER overpotentials of these three Cu/MoP composites are 233 mV (Cu/MoP-5), 265 mV (Cu/MoP), and 315 mV (Cu/MoP-15), respectively. The decreasing HER activity in the order of Cu/MoP-5 > Cu/MoP > Cu/MoP-15 indicates that MoP plays a key role in the water dissociation behavior of Cu/MoP, which produces active hydrogen. As presented in Supplementary Figure S4b, these three Cu/MoP composites have obviously enhanced current response in the mixed solution of 1.0 M KOH and 1.0 NaNO_3_, and Cu/MoP has the best NITRR catalytic activity, while Cu/MoP-15 shows the worst catalytic activity, indicating the excessive copper content can lead to bad NITRR activity.

To further investigate the catalytic activity and selectivity of the NITRR on the as-prepared catalysts, the resultant solution after the NITRR at different applied potentials for 30 min was collected (Figure 3c), the indophenol blue method was utilized to assess the concentrations of NH_3_. For the Cu/MoP composite, the product solution stained with the indophenol blue indicator is shown in the inset of Figure 3d, and the corresponding UV-visible absorption curves are presented in Figure 3d. With an increase in the given potential, the color of the product solution becomes darker and the corresponding light absorption intensity increases, indicating that the NH_3_ concentration increases. According to the calibration of the NH_3_ standard curve (Supplementary Figure S5), the highest Faraday efficiency of NH_3_ conversion is 98.9% and is obtained at −0.5 V, and the corresponding NH_3_ yield rate is 30.72 mmol h^−1^ cm^−2^, indicating the excellent NITRR catalytic activity of the Cu/MoP composite. In addition, the UV-visible absorption curves of NO_2_^−^ and N_2_H_4_ in the product are shown in Supplementary Figure S8, where the Faraday efficiency of NO_2_^−^ at −0.5 V voltage is 0.98% (Supplementary Figure S8c), according to the calibration of the NO_2_^−^ standard curve (Supplementary Figure S6), while N_2_H_4_ can hardly be detected by the Watt–Chrisp method, indicating its low yield. The high NITRR catalytic activity and NH_3_ FEs of the as-prepared Cu/MoP surpass those of most recently reported advanced catalyst for the NITRR (Supplementary Table S1), suggesting the great potential application of Cu/MoP for nitrate-to-ammonia conversion.

For comparison, the products of Cu and MoP were also evaluated using the same method. As shown in Supplementary Figure S9, the highest Faraday efficiency of ammonia production of the isolated Cu nanoparticles is 76.8% at a voltage of −0.3 V, but its yield is only 4.30 mmol h^−1^ cm^−2^, and it also shows a high Faraday efficiency of NO_2_^−^ production at this voltage. Meanwhile, NH_3_ faraday efficiency of the isolated Cu sample is 52.56% at −0.5 V (vs. RHE), while the corresponding NO_2_^−^ faraday efficiency is 0.98% and 22.2% at the same potential. Therefore, a dramatic increase in NH_3_ selectivity and a significant decrease in byproduct NO_2_^−^ of the Cu/MoP composite reveal high-efficiency conversion of NO_3_^−^ to NH_3_, which requires effective assistance of sufficient active hydrogen and efficient N-intermediate conversion. In addition, the Faraday efficiency of ammonia close to 100% indicates that hydrogen evolution on the Cu/MoP complex is inhibited, and the generated active hydrogen mainly participates in nitrate-to-ammonia conversion. For MoP, the highest Faraday efficiency of ammonia production is 60.34% at −0.3 V, but its yield is only 1.44 mmol h^−1^ cm^−2^ (Supplementary Figure S9). Therefore, both Cu and MoP exhibit lower NITRR ammonia production efficiency and yield, while Cu nanoparticles coupled with MoP exhibit enhanced NITRR catalytic activity and high ammonia production selectivity. According to the electrochemical HER and NITRR results, MoP nanoparticles can effectively promote water decomposition to produce protons, which are easily released in the form of H_2_ on the isolated MoP nanoparticles. Cu nanoparticles can provide abundant active sites for the NITRR process, but the conversion efficiency from NO_3_^−^ to NH_3_ is limited due to the lack of active hydrogen. For the Cu/MoP composite, protons produced by the MoP phase in Cu/MoP may be more inclined to participate in the reduction conversion from NO_3_^−^ to NH_3_ on the surface of Cu nanoparticles. Therefore, the synergic catalysis of Cu and MoP can significantly improve the NITRR catalytic activity. To confirm that NH_3_ produced originates from the NITRR rather than nitrogen-containing contaminants, a^15^N isotope labeling experiment was performed for the Cu/MoP (Figure 3f). With ^14^NO_3_^−^ as the N source, the ^1^H-NMR spectrum presents a triple peak with the coupling constant of J_N-H_ = 52.4 Hz, which is ascribed to the standard ^14^NH_4_^+^. When fed with ^15^NO_3_^−^, a doublet with a coupling constant of J_N-H_ = 72 Hz is detected (corresponding to the characteristic peak of ^15^NH_4_^+^) (Zhang et al., 2022). This result confirms that the generation of NH_3_ is from the NITRR.

The underlying mechanism of remarkable NITRR activity of Cu/MoP was further explored by assessing electrochemical impedance spectroscopy (EIS) and electrochemical active surface area (ECSA). Figure 3g shows the corresponding Nyquist plots, and the similar shape of these three samples suggests that all samples undergo similar charge transfer processes. Furthermore, an equivalent circuit (inset of Figure 3g) was used to simulate the Nyquist plots, which consist of solution resistance (*R*_s_), charge transfer resistance (*R*_ct_), and constant phase element (CPE). According to the fitting results, Cu/MoP exhibits a smaller *R*_ct_ value (7.8 Ω) than Cu (17.4 Ω) and MoP (20.2 Ω), indicating that coupling Cu with MoP is favorable for reducing charge-transport impedance and accelerating the electron transport rate. As shown in Supplementary Figure S11, the *R*_ct_ value of Cu/MoP is also smaller than that of Cu/MoP-5 (10.8 Ω) and Cu/MoP-15 (12.5 Ω), which may be attributed to more Cu and MoP interfaces in the Cu/MoP composite. The cyclic voltammetry (CV) curves of the as-obtained samples were recorded at various scan speeds between 10 and 110 mV s^−1^ within the non-faradic range (Supplementary Figure S12), which were used to calculate the double-layer capacitance (*C*_dl_) and assess the ECSA. As illustrated in Supplementary Figure S12f, the *C*_dl_ value of Cu/MoP is 27.01 mF cm^−2^, larger than that of MoP (0.60 mF cm^−2^), Cu (1.07 mF cm^−2^), Cu/MoP-5 (23.79 mF cm^−2^), and Cu/MoP-15 (19.47 mF cm^−2^), suggesting that the Cu/MoP composite has more potential active sites for the NITRR. Fast charge transport rate and abundant active sites result from enriched Cu and MoP interfaces and their induced modulation for electronic structures in the Cu/MoP composite. A long-term NITRR stability test was performed to evaluate the catalytic robustness of Cu/MoP under a high NH_3_ production rate. Figure 3h shows the chronoamperometry curve of Cu/MoP in the mixed solution of 1.0 M KOH and 1.0 M NaNO_3_ at −0.5 V. As observed, the initial current density is recorded at 406 mA cm^–2^. The reduction current density steadily decreases as the reaction time extends to 8 h, but it returns to the initial current density value upon adding fresh electrolyte, suggesting that the decrease is due to the rapid depletion of NO_3_^−^. This result indicates that the Cu/MoP composite exhibits robust catalytic activity. NH_3_ FE during the long-term operation was also evaluated by assessing the NH_3_ concentration of the product solution at 1, 2, 4, 8, and 10 h. According to the UV-Vis absorption spectrum and the calibration curve (Supplementary Figure S13), the NH_3_ FE keeps remains nearly unchanged, implying good durability of the Cu/MoP catalyst for the NITRR.

**FIGURE 4:**
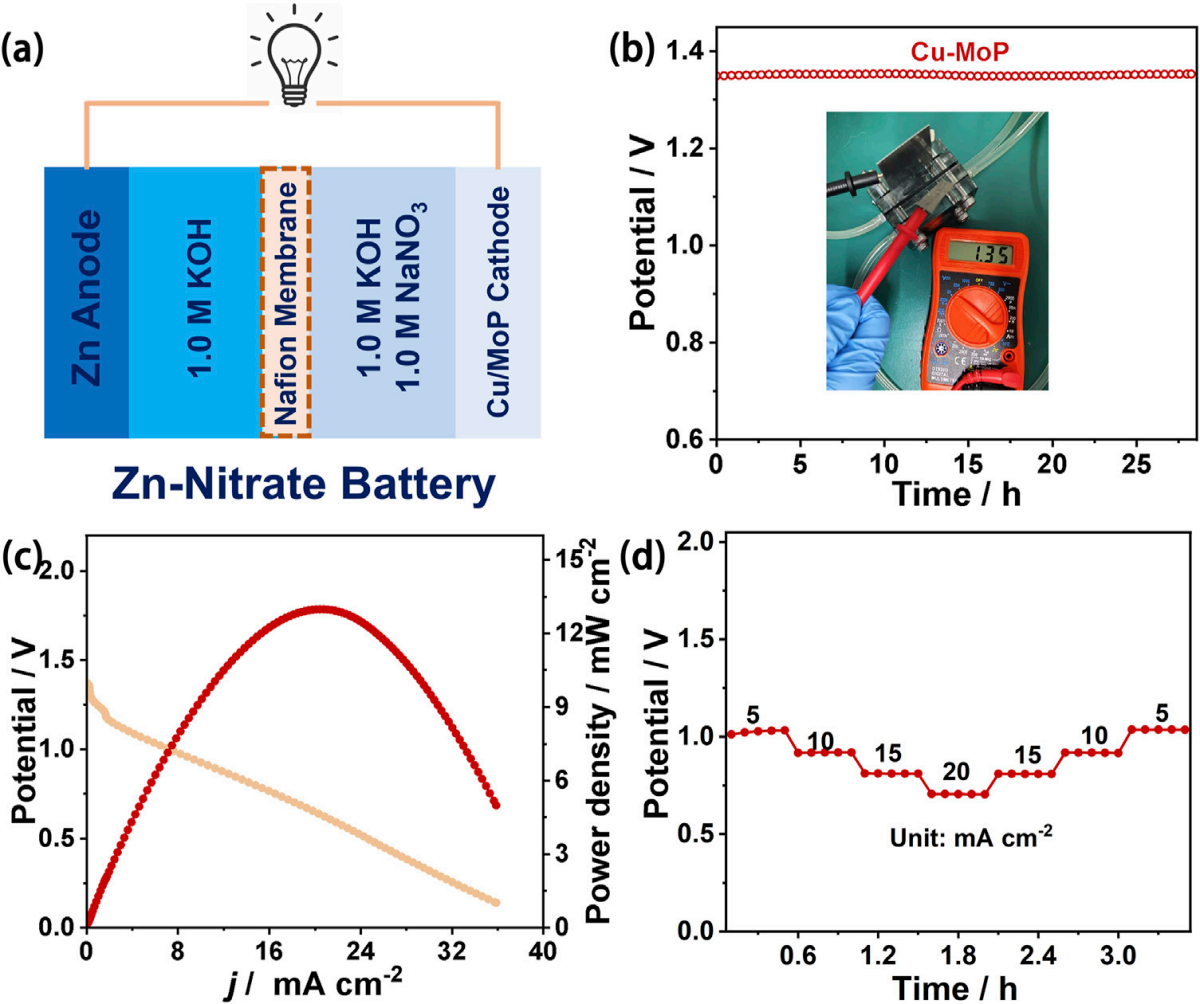
Zinc nitrate battery performance evaluation of Cu/MoP. **(a)** Schematic demonstration of the Zn-NO_3_^−^ battery with the Zn anode and the Cu/MoP cathode; **(b)** open-circuit voltage (the inset is the OCV tested using the potentiometer); **(c)** discharge curve and the corresponding output power density curve; and **(d)** rate capability.

Considering the excellent electrochemical NITRR performance of the Cu/MoP composite, we further assembled a zinc-nitrate (Zn-NO_3_^−^) battery using Cu/MoP as the cathode and a Zn plate as the anode, and the cathode electrolyte consisted of a mixed solution of 1.0 M KOH and 1.0 M NaNO_3_ aqueous solution, while 1.0 M KOH was employed as the anode electrolyte. The two compartments were separated by a Nafion membrane. The schematic diagram of the battery device is shown in Figure 4a. A “killing three birds with one stone” capability can be achieved by this Zn-NO_3_^−^ battery, enabling simultaneous energy supply, ammonia production, and nitrate pollutant removal. As shown in Figure 4b, this Zn-NO_3_^−^ battery delivers an open-circuit voltage of 1.35 V, and the output voltage remains stable during more than 25 h of testing. The battery performance was also evaluated usinf power density. The discharge curve was recorded at a scan rate of 5 mVs^−1^ (Figure 4c). The corresponding power density was calculated using the equation P = I × V (Zhou and Sun, 2022), and the peak power density of this Cu/MoP-based battery reaches 12.97 mW cm^−2^, which exceeds that of recently reported noble metal and non-noble metal-based NITRR catalysts. Furthermore, the discharge performance was also assessed at different current densities. As presented in Figure 4d, the voltages at different discharge current densities from 5 to 20 mA cm^−2^ remain stable, indicating remarkable rate stability. These results indicate that Cu/MoP-based Zn-NO_3_^−^ battery delivers excellent battery performance, showing good potential for practical application.

## 3 Conclusion

This study presents the synthesis and application of a Cu/MoP composite catalyst for enhanced electrochemical NITRR and excellent zinc-nitrate battery performance. The integration of Cu and MoP in the composite optimizes electronic structure and produces the electron-deficient state of Cu and the electron-rich state of MoP. Thus, the NITRR process and the generation of active hydrogen were synergistically optimized. The Cu/MoP composite, prepared via one-pot calcination, exhibits superior NITRR activity, achieving a high NH_3_ Faradaic efficiency of 98.9%. Employed as a cathode catalyst for the zinc-nitrate battery, this composite shows robust stability and high power density. This work reveals the potential of Cu/MoP composites for sustainable ammonia production and energy storage applications, offering an efficient solution to current challenges in the electrochemical NITRR of Cu-based catalysts.

## 4 Materials and methods

### 4.1 Chemicals

Ammonium dihydrogen phosphate (NH_4_H_2_PO_4_, >98%), ammonium molybdate tetrahydrate ((NH_4_)_6_Mo_7_O_24_⋅4H_2_O, 99%), copper acetate (Cu(Ac)_2_, 98%), polyacrylonitrile (PAN), sodium nitrate (NaNO_3_, 99.99%), potassium hydroxide (KOH, >98%), sodium nitrate −15N (Na^15^NO_3_, >99.0%), deuterium oxide (D_2_O, 99.9 at.% D), sodium nitrite (NaNO_2_, 99%), sodium hydroxide (NaOH, >98%), salicylic acid (C_7_H_6_O_3_, 99%), sodium nitroferricyanide dehydrate (C_5_FeN_6_Na_2_O·2H_2_O, 99%), sodium hypochlorite solution (NaClO, available chlorine 4.0%), hydrochloric acid (HCl, 37%), and N-(1-naphthyl) ethylenediamine dihydrochloride (C_12_H_14_N_2_·2HCl, 98%) were bought from Aladdin Reagent. Nafion solution (5 wt.%) was obtained from DuPont. All reagents were directly used without further purification.

### 4.2 Synthesis of molybdenum phosphide-coupled copper nanoparticles, molybdenum phosphide, and copper nanoparticles

Molybdenum phosphide-coupled copper nanoparticles were prepared by a facile one-pot high-temperature pyrolysis approach. Typically, 1 mmol (NH_4_)_6_Mo_7_O_24_⋅4H_2_O, 8 mmol NH_4_H_2_PO_4_, 3.25 g PAN, and a certain molar equivalent of Cu(Ac)_2_ (5, 10, and 15 mmol) were thoroughly ground into a homogeneous powder, which was then calcinated at 800°C in H_2_/Ar (10%) for 2 h. Three products were finally obtained and noted as Cu/MoP-5, Cu/MoP, and Cu/MoP-15. The isolated MoP and Cu were synthesized according to the same procedure as Cu/MoP, but the former without Cu(Ac)_2_ and the latter without (NH_4_)_6_Mo_7_O_24_⋅4H_2_O and NH_4_H_2_PO_4_.

### 4.3 Material characterization

X-ray diffraction (XRD) tests were performed on a Bruker D8 Advance with Cu Kα radiation under a voltage of 40 kV and a current of 40 mA. X-ray photoelectron spectroscopy (XPS) measurements were conducted on Thermo Scientific-ESCALAB Xi+. Raman results were collected on Thermo DXR2xi. The morphologies and nanostructures of the as-obtained catalysts were investigated using a field emission scanning electron microscope (ZEISS Sigma 500) and a transmission electron microscope coupled with an energy dispersive spectrometer (Thermo Fisher Scientific Talos F200S). ^1^H-NMR measurements were performed on a Bruker 400 MHz ASCEND ADVANCE III HD (nuclear magnetic resonance system, NMR-400). UV-Vis spectroscopy measurements were carried out using a UV-Vis Spectrometer Lambda 2S.

### 4.4 Electrochemical measurements

All electrochemical tests were conducted on CHI-760E electrochemistry workstation using a three-electrode electrochemical system at room temperature, and the mixed solution containing 1.0 M KOH and 1.0 NaNO_3_ was used as the electrolyte for NITRR processes, while 1.0 M KOH aqueous solution was used as the media for the comparison. The glassy carbon electrode (GCE, diameter = 3 mm) loaded with the as-synthesized catalysts, graphite rod, and Hg/HgO electrode, were employed as the working electrodes, counter electrode, and reference electrode, respectively. All potentials in this work were converted to the reversible hydrogen electrode (RHE) using the equation *E*(RHE) = *E*(Hg/HgO) + 0.098 + 0.059 × pH.

In a typical procedure for the preparation of the working electrode, 5 mg of catalyst powder was ultrasonically dispersed in a mixture containing 20 μL of Nafion and 480 μL of ethanol. Then, 7 μL of the resulting ink was drop-cast onto the GCE, and the loading mass of the catalyst was 1.0 mg cm^−2^. The LSV curves were recorded with a scanning speed of 5 mV s^−1^ in a typical H-type cell (containing 50 mL electrolyte) separated by a membrane (Nafion 117), and the corresponding Tafel plots can be derived. Chronoamperometry measurements at a series of applied potentials were carried out to calculate the yield and Faraday efficiency (FE) of ammonium and nitrite, while the same test at the constant potential of −0.4 V (vs. RHE) was performed to assess the long-term stability. Electrochemical impedance spectroscopy (EIS) measurements were carried out at – 0.15 V with an AC amplitude of 5 mV in the frequency range of 100 kHz to 0.1 Hz. CV tests with different scanning speeds from 10 to 110 mV/s were performed to collect the double-layer capacitance in the non-Faraday potential range of 0.45–0.55 V (vs. Hg/HgO). For Na^15^NO_3_ isotope labeling experiments, a 20 mL solution containing 1.0 M KOH +50 mM Na^15^NO_3_ was utilized as the NITRR electrolyte. Then, 0.05 mL of D_2_O and 0.45 mL of diluted electrolyte were added into a nuclear magnetic tube for further NMR (400 MHz) test.

### 4.5 Assembly of the zinc-nitrate battery and electrochemical test

For the zinc-nitrate battery, hydrophobic carbon paper-supported Cu, N-MoP catalyst (loading mass of 1.0 mg cm^−2^) was used as the cathode, and a Zn plate with a diameter of 3 cm was used as the anode. The cathode electrolyte consisted of a mixed solution containing 1.0 M KOH and 1.0 M NaNO_3_, while 1.0 M KOH solution was used as the anode electrolyte. The two compartments were separated by a bipolar membrane. Discharge polarization curves (at a scan rate of 5 mV s^−1^), open-circuit potential measurements, and galvanostatic tests were conducted using Land 2001A battery testing system on a custom-built battery device at room temperature, respectively.

### 4.6 Determination of ammonia

The amount of NH_3_ produced was quantified using the indophenol blue method. Typically, 1.0 mL of electrolyte after NITRR tests was collected and diluted 20 times with the electrolyte. Then, 1.25 mL of a mixed solution containing 0.625 M NaOH, 0.36 M salicylic acid, and 0.17 M sodium citrate was added into the diluted sample. After that, 150 μL of sodium nitroferricyanide solution (10 mg/mL) and 75 μL NaClO solution (available chlorine 4.0 wt%) were added. The UV-Vis absorption spectrum was recorded after the solution was allowed to stand for 2 h under ambient conditions, and the absorbance was recorded at a wavelength of 658 nm. A calibration curve correlating ammonium concentration to absorbance was built using standard NH^4+^ solutions of known concentrations prepared from NH_4_Cl in 1.0 M KOH.

### 4.7 Colorimetric detection of $NO_{2}^{-}$

A volume of 0.10 mL of 2.0 M HCl solution was added to 5 mL of the diluted electrolyte. Then, 0.10 mL of N-(1-naphthyl) ethylenediamine dihydrochloride solution (10 mg/mL) was added to the mixture. After standing for 0.5 h, the absorbance value of $NO_{2}^{-}$ was obtained between 450 nm and 600 nm. A standard calibration curve was also obtained using known concentrations of NaNO_2_ in 1.0 M KOH.

### 4.8 Na^15^NO_3_ isotope labeling experiments

The Na^15^NO_3_ reduction tests were performed using Na^15^NO_3_ as the nitrate source instead of NaNO_3_, while the electrochemical tests followed the same method described above. The pH value of the resulting electrolyte was adjusted to 3 using 2 M H_2_SO_4_. A mixed solution containing 500 μL of the electrolyte and 50 μL of D_2_O was prepared for NMR (400 MHz) detection. For comparison, the electrolyte produced by $NO_{3}^{-}$ reduction was also analyzed under the same conditions.

### 4.9 Ammonia yield rate and faradaic efficiency

Yield rates and FEs of NH_3_ and $NO_{2}^{-}$ were calculated using the following equation:

$$\text{FE }\left({\text{NH}}_{3}\right)=\left(8F\times C\times V\times n\right)\;/\;Q,$$

$$\text{Yield rate }\left({\text{NH}}_{3}\right)=\left(C\times V\times n\right)\;/\;\left(t\times A\right),$$

$$\text{FE }\left(NO_{2}^{-}\right)=\left(2F\times C\times V\times n\right)/Q,$$

$$\text{Yield rate}(NO_{2}^{-})=\left(C\times V\times n\right)/\left(t\times A\right),$$

where *C* is the obtained NH_3_ concentration, *F* is the Faraday constant (96,485 C/mol), *V* is the volume of the electrolyte, *n* corresponds to the dilution factor, *Q* is the total charge passed through the electrode surface, and *A* is the geometer area of the working electrode (0.07 cm^2^).

### 4.10 Assembly of the zinc-nitrate battery and electrochemical test

The Cu/MoP catalyst-modified carbon cloth (with a loading mass of 1.0 mg cm^−2^) and a Zn plate (2.0 cm^2^) were used as the cathode and anode, respectively, for the zinc-nitrate battery. A custom-made battery device with separate cathode and anode chambers was employed for electrochemical tests. Typically, a mixed solution of 1.0 M KOH and 1.0 M NaNO_3_ was used as the cathode electrolyte, while 1.0 M KOH was used as anode electrolyte. These two compartments were separated by a Nafion membrane (Nafion 117). Discharge polarization curves were recorded at a scan rate of 5 mV/s using a CHI 760E workstation, and galvanostatic tests were performed using the Land 2001A battery testing system. The power density (*P*) of the zinc-nitrate battery was calculated using the equation *P* = *I* × *V*, where *I* and *V* are the discharge current density and voltage, respectively.

## Data Availability

The original contributions presented in the study are included in the article/Supplementary Material; further inquiries can be directed to the corresponding authors.
