# Supplementary material for: Strongly coupling Cu with MoP for high-efficiency electrochemical nitrate-to-ammonia conversion and zinc-nitrate battery applications
Source: Front Chem. 2025 Jul 17;13:1629904. doi: 10.3389/fchem.2025.1629904 (PMC12310678; doi:10.3389/fchem.2025.1629904)
Supplement: Supplementary file 1 [file DataSheet1.docx]

**Strongly Coupling Cu with MoP for High-efficiency Eletrochemical Nitrate-to-Ammonia Conversion and Zinc-Nitrate battery**

**Chen Yang ^1^, Yuanyuan Chen ^1^, Zhimin He ^1,^*, Rong Li ^1,^* and Xinglong Gou ^2,^***

^1^ Precise Synthesis and Function Development Key Laboratory of Sichuan Province, College of Chemistry and Chemical Engineering, China West Normal University, Nanchong 637000, China

^2^ Sichuan University of Arts and Science, Dazhou, 635002, China

*Corresponding authors. E-mail: [hezhimin@cwnu.edu.cn](mailto:hezhimin@cwnu.edu.cn) (Z. He), [lirong406@126.com](mailto:lirong406@126.com) (R. Li), [gouxlr@126.com](mailto:gouxlr@126.com) (X. Gou)


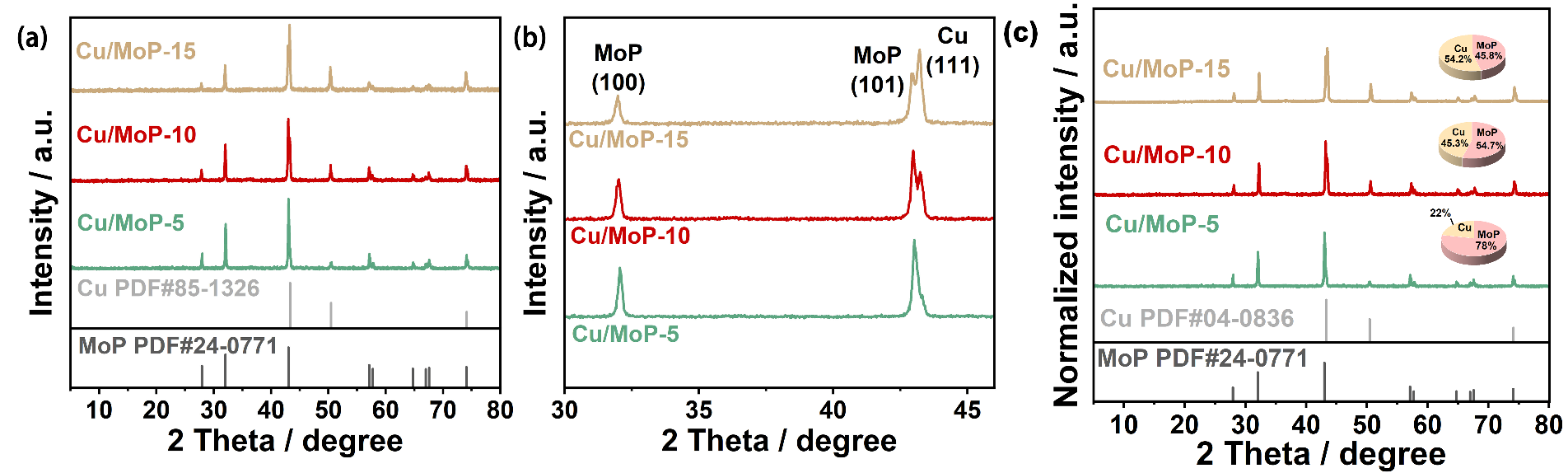


**Figure S1** (a) XRD patterns of Cu/MoP-5, Cu/MoP-10 and Cu/MoP-15; (b) the partially enlarged diffraction peak; (c) Normalized XRD patterns of Cu/MoP-5, Cu/MoP-10 and Cu/MoP-15.


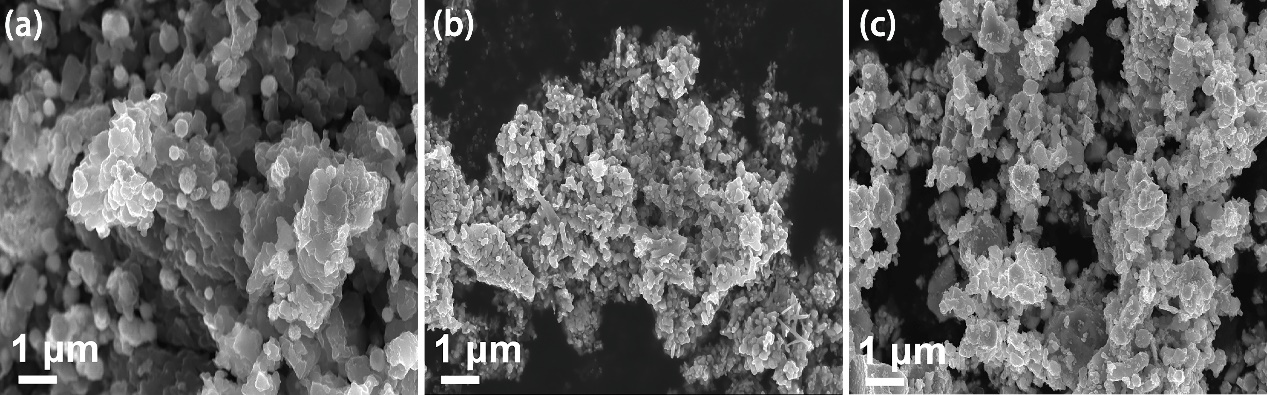


**Figure S2** FE-SEM images of (a) Cu, (b) MoP and (c) Cu/MoP.


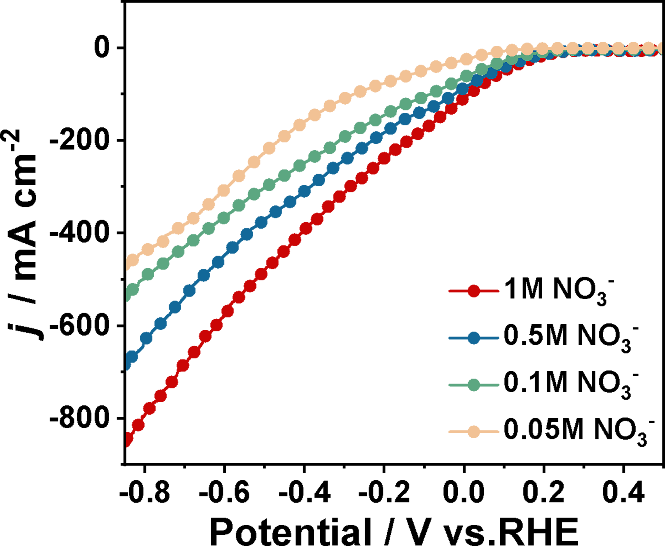


**Figure S3** LSV curves of Cu/MoP in electrolyte with different NO‒ 3 concentrations.


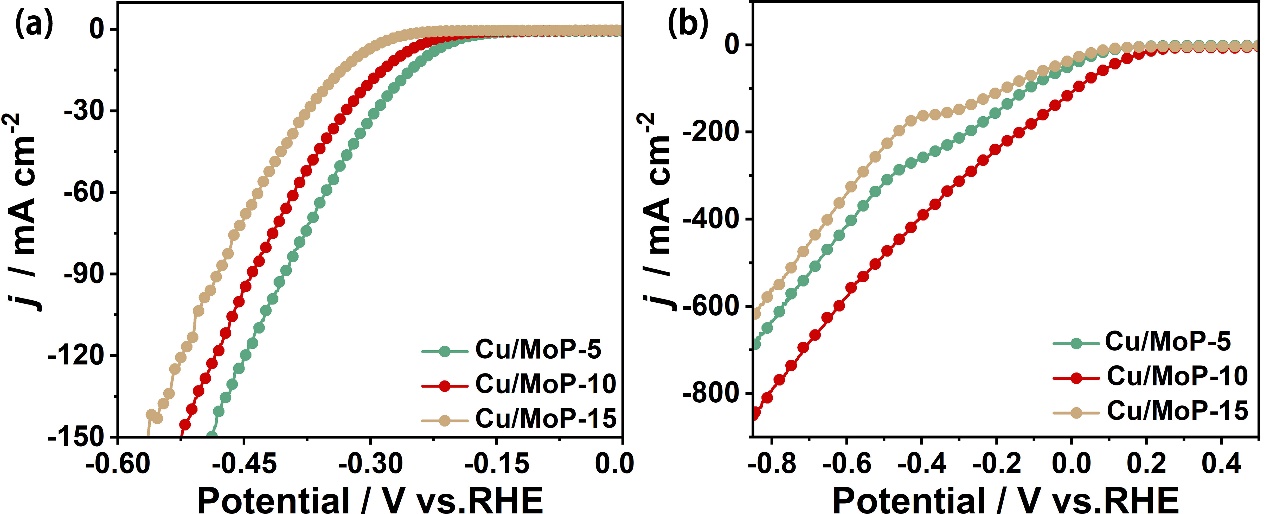


**Figure S4** LSV curves of different Cu/MoP composites in (a) 1.0 M KOH and (b) the mixed electrolyte of 1.0 M KOH and 1.0 NaNO_3_


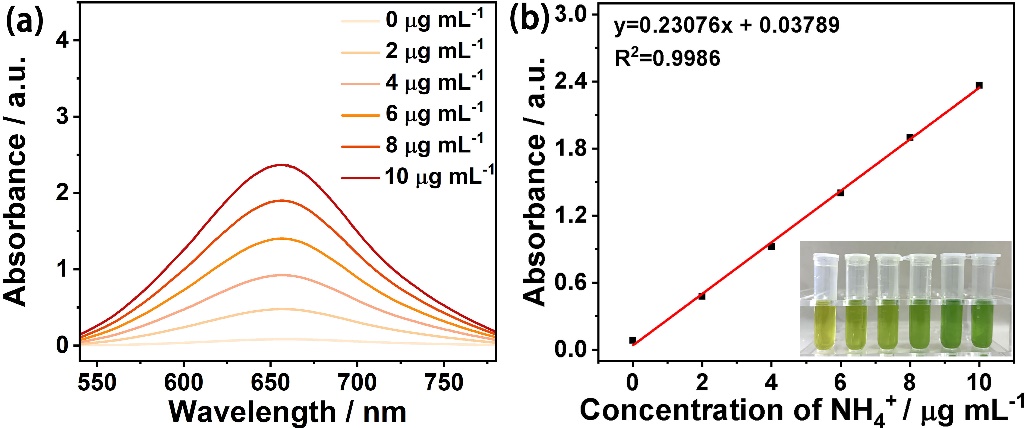


**Figure S5** (a) The UV-Vis adsorption curves and (b) calibration curves of the electrolyte with the given concentrations of NH_4_Cl.


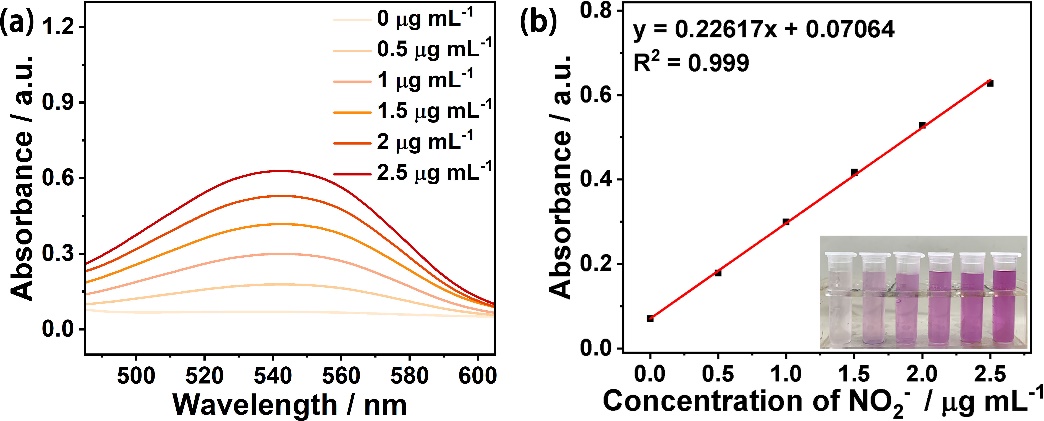


**Figure S6** (a) The UV-Vis adsorption curves and (b) calibration curves of the electrolyte with the given concentrations of NaNO_2_.


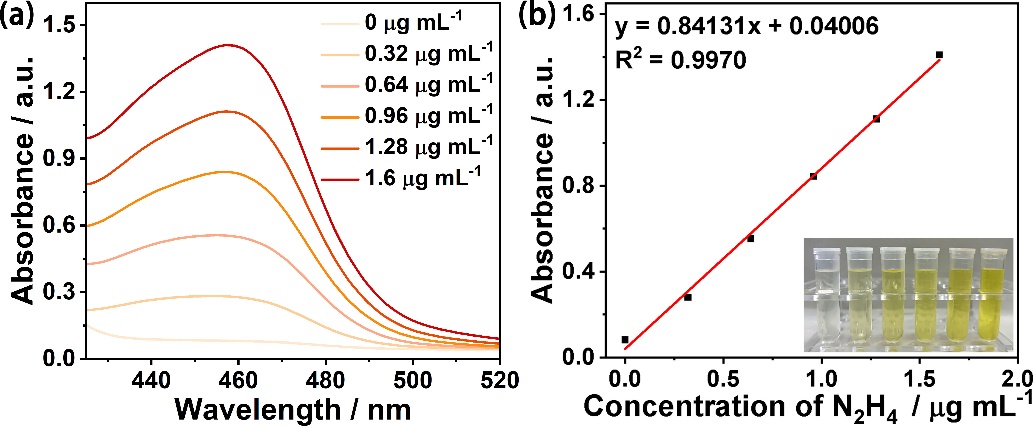


**Figure S7** (a) The UV-Vis adsorption curves and (b) calibration curves of the electrolyte with the given concentrations of N_2_H_4_.


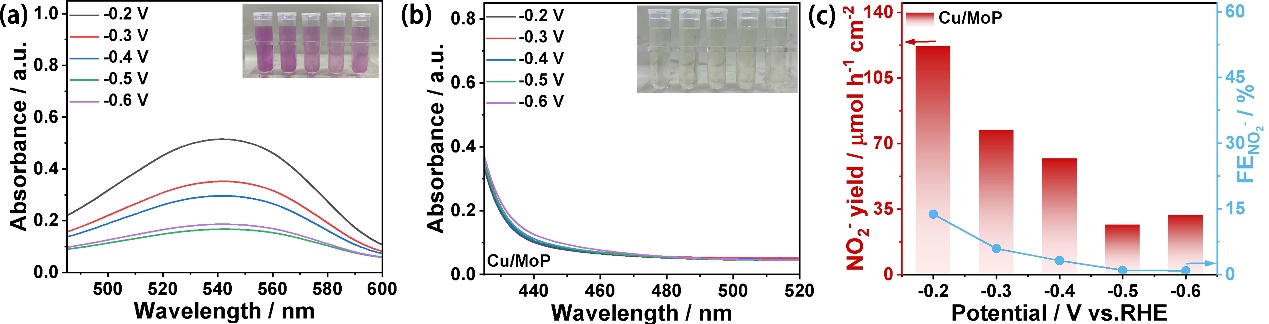


**Figure S8** The UV-Vis adsorption spectra of the product solution for (a) NO‒ 2 and (b) N2H4 after chronoamperometry tests of Cu/MoP (the inset is the digital photograph of product solution stained with indicator); (c) the corresponding FEs and yield rates of NO‒ 2.


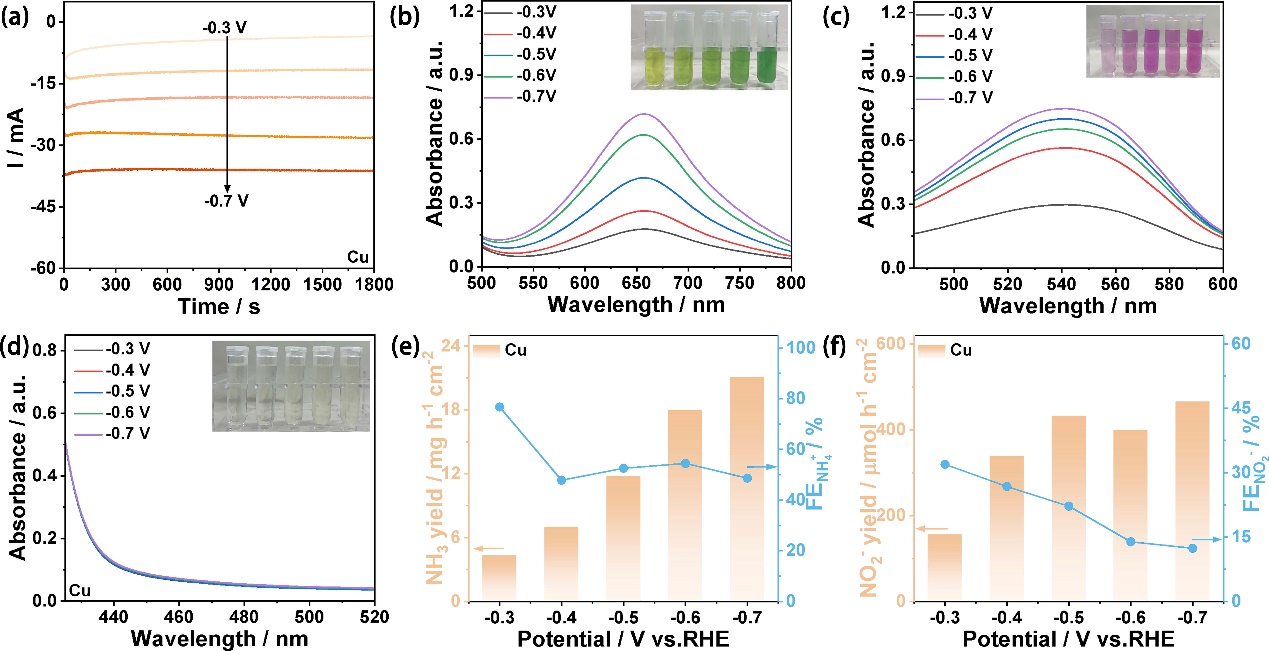


**Figure S9** (a) chronoamperometry tests of the isolated Cu sample for 0.5 h at different applied potentials from -0.3 to -0.7 V; UV-vis absorption spectra of the product solution for (b) NH_3_, (c) NO‒ 2 and (d) N_2_H_4_ after chronoamperometry tests of Cu (the inset is the digital photograph of product solution stained with indicator); FEs and yield rates of (e) NH_3_ and (f) NO‒ 2 for Cu samples.


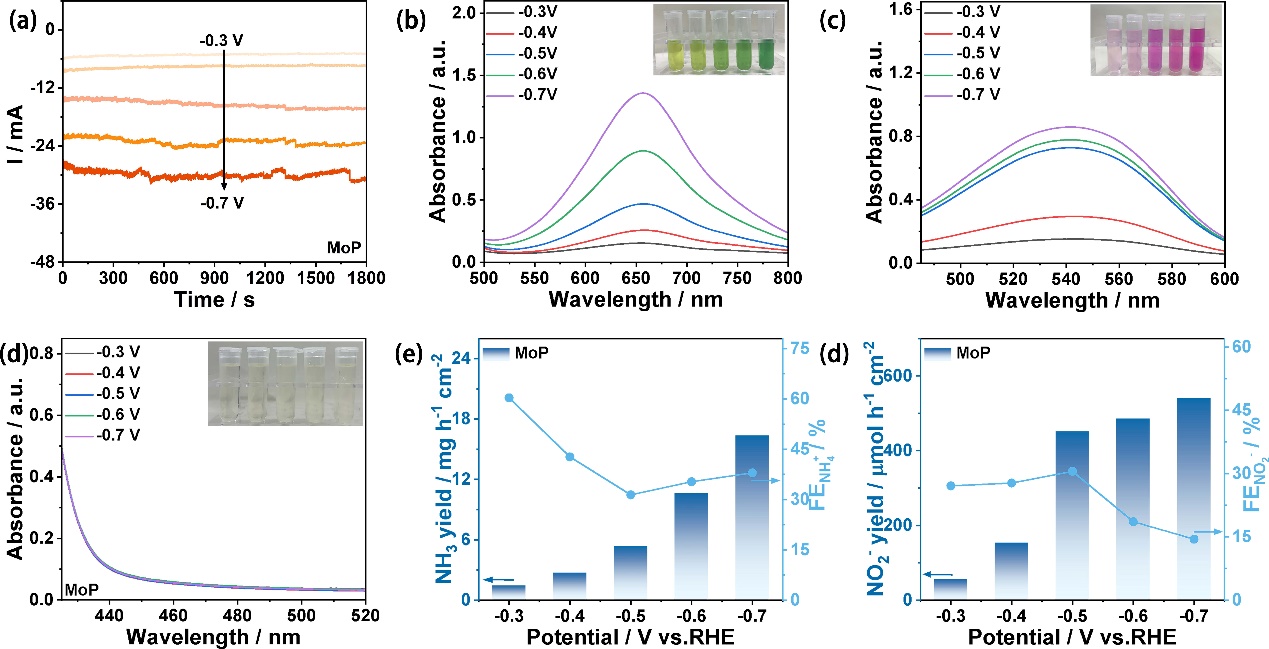


**Figure S10** (a) chronoamperometry tests of the isolated MoP sample for 0.5 h at different applied potentials from -0.3 to -0.7 V; UV-vis absorption spectra of the product solution for (b) NH_3_, (c) NO‒ 2 and (d) N_2_H_4_ after chronoamperometry tests of MoP (the inset is the digital photograph of product solution stained with indicator); FEs and yield rates of (e) NH_3_ and (f) NO‒ 2 for MoP samples.


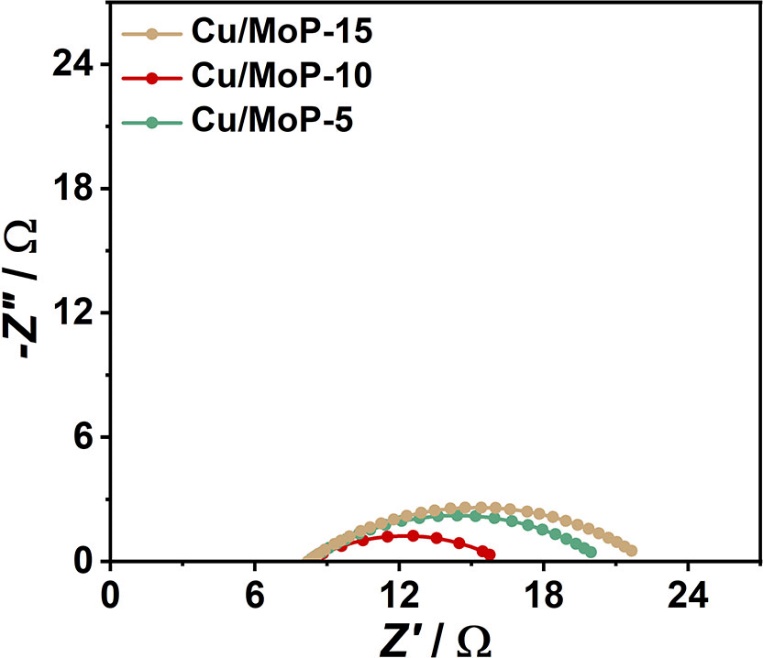


**Figure S11** Nyquist plots of different Cu/MoP samples.


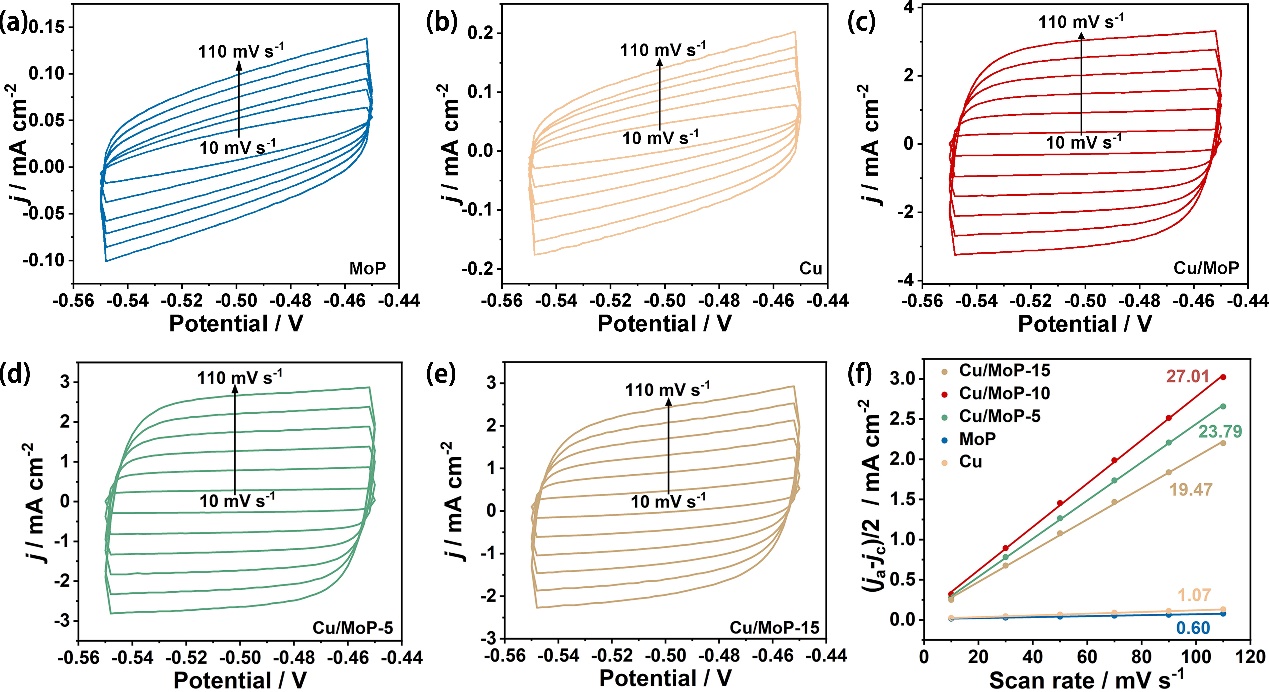


**Figure S12** CV curves of (a) MoP, (b) Cu, (c) Cu/MoP, (d) Cu/MoP-5 and (c) Cu/MoP-15 at different scan rates from 10 to 110 mV s^-1^ within the non-Faradaic region from -0.45 to -0.55 V (vs. Hg/HgO); (f) the corresponding scanning speed dependance of capacitive current at -0.5 V (vs. Hg/HgO).


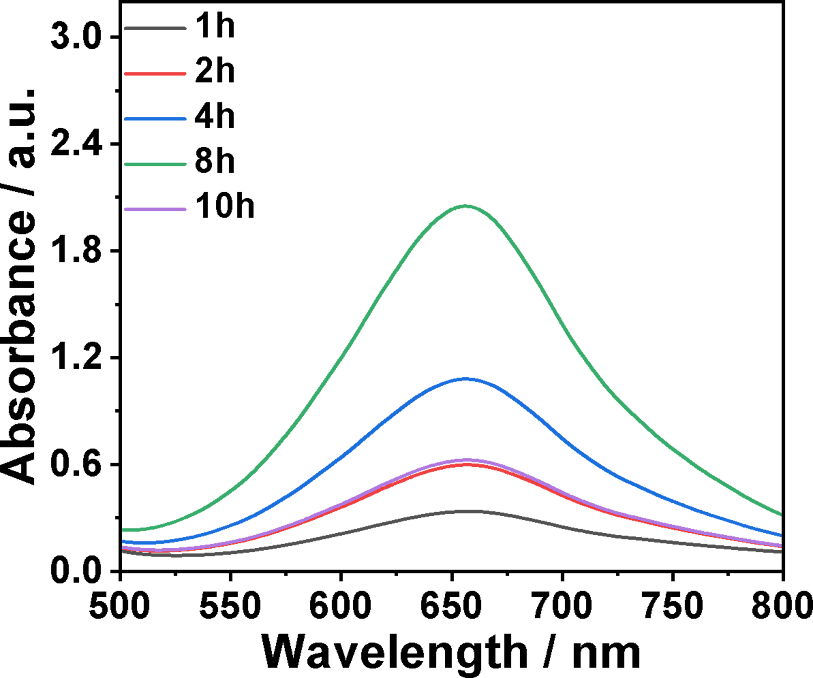


**Figure S13** UV-vis absorption spectra of the product solution at different reaction time during long-term i-t measurement.

**Table S1 Comparison of NH3 FEs and yield rate of Cu/MoP with recently reported NITRR catalysts**

| **Catalysts** | **Electrolyte** | **FE (%)** | | | **NH_3_ yield rate** | **Reference** |
| --- | --- | --- | --- | --- | --- | --- |
| Cu/MoP | 1 M KOH  （$\text{1 M NO}_{3}^{-}$） | | 98.9 | 30.72  mg h^-1^ cm^-2^ | | This work |
| Ag/Cu/MXene | 1 M KOH  （$\text{1 M NO}_{3}^{-}$） | | 87.7 | 10.3  mol g -1 cat. h^-1^ | | Catal. Sci. Technol. 2025 |
| Ru-Co CHNWS | 1 M KOH  （$\text{1 M NO}_{3}^{-}$） | | 99 | 8.21  mg h^-1^ cm^-2^ | | Chem. Eng. J. 2024, 490, 151883 |
| Ni-MOF-Ru | 1 M KOH  （$\text{1 M NO}_{3}^{-}$） | | 91.5 | 1.31  mmol h^-1^ cm^-2^ | | ACS Catal. 2024, 14, 16205-16213 |
| SnAg | 1 M KOH  （$\text{1 M NO}_{3}^{-}$） | | 94.5 | 78.6  mg h^-1^ cm^-2^ | | Angew. Chem. Int. Ed. 2024, 63, e202410251 |
| Fe-BCN | 1 M KOH  （$\text{0.5 M NO}_{3}^{-}$） | | 97.48 | 2.17  mg h^-1^ cm^-2^ | | ACS Appl. Nano Mater. 2024, 7, 14654−14664 |
| CoO NC/Graphene | 1 M KOH  （$\text{1 M NO}_{3}^{-}$） | | 99 | 25.63  mg h^-1^ cm^-2^ | | Adv. Energy Mater , 2023, 13, 2204236. |
| Ni nanoparticles | 1 M KOH  （$\text{1 M NO}_{3}^{-}$） | | 93 | 15.49  mmol h^-1^ cm^-2^ | | Energy Environ. Sci. 2023,16, 2611-2620 |
| MoO_2_/Fe_4_N/C | 1 M KOH  （$\text{1 M NO}_{3}^{-}$） | | 99.3 | 1.67  mmol h^-1^ cm^-2^ | | Nanoscale, 2023, 15,  14439-14447 |
| Ni_3_Co_6_S_8_ | 1 M KOH  （$\text{0.5 M NO}_{3}^{-}$） | | 85.3 | 140.5  μmol h^-1^ cm^-2^ | | Appl. Catal. B: Environ. 2023, 3324, 122193 |
| Co_3_D nanoarray | 1 M KOH  （$\text{2 M NO}_{3}^{-}$） | | 86.2 | 68.4  mg h^-1^ cm^-2^ | | Nat. Commun. 2023, 14, 1619 |
| Au/Cu | 1 M KOH  （$\text{0.5 M NO}_{3}^{-}$） | | 98 | 73.4  mg h^-1^ cm^-2^ | | Small Struct. 2023, 2200308 |
| Ru-CuNW | 1 M KOH  （$\text{0.32 M NO}_{3}^{-}$） | | 96 | 76.89  mg h^-1^ cm^-2^ | | Nat. Nanotechnol. 2022, 17, 759-767 |
| Gd_SA_-D-NiO_400_ | 1 M KOH  （$\text{1 M NO}_{3}^{-}$） | | 97 | 36.9  mmol g^-1^ h^-1^ | | ACS Nano. 2022, 16, 15297-15309 |
| Cu SACs | 1 M KOH  （$\text{1 M NO}_{3}^{-}$） | | 95.5 | 13.8  mol g^-1^ h^-1^ | | ChemSusChem, 2022, 15, e202200231 |
